# Microbiology neutralization of zearalenone using *Lactococcus lactis* and *Bifidobacterium* sp.

**DOI:** 10.1007/s00216-017-0555-8

**Published:** 2017-08-29

**Authors:** A. Król, P. Pomastowski, K. Rafińska, V. Railean-Plugaru, J. Walczak, B. Buszewski

**Affiliations:** Faculty of Chemistry, Centre for Modern Interdisciplinary Technologies, Nicolaus Copernicus University, 7 Gagarina Str, 87-100 Torun, Poland

**Keywords:** Zearalenone, Toxicity, Neutralization, Lactic acid bacteria

## Abstract

The aim of the study was to neutralize zearalenone by lactic acid bacteria (LAB) such as *Lactococcus lactis* and *Bifidobacterium* sp*.* and investigate the mechanism of zearalenone (ZEA) binding. Neutralization of ZEA by LAB was confirmed by identification of binding kinetics and spectroscopic studies such as Fourier transform infrared spectroscopy (FT-IR) and matrix-assisted laser desorption/ionization time-of-flight mass spectrometry (MALDI-TOF-MS). The obtained results showed that the kinetic process of zearalenone binding to *L. lactis* is not homogeneous but is expressed with an initial rapid stage with about 90% of ZEA biosorption and with a much slower second step. In case of *Bifidobacterium* sp*.*, the neutralization process is homogeneous; the main stage can be described with about 88% of ZEA biosorption. MALDI–TOF-MS measurements and FTIR analysis confirmed the uptake of zearalenone molecules by bacterial species. Moreover, the assessment of dead and live lactic acid bacteria cells after zearalenone treatment was performed using fluorescence microscopy.

**Graphical abstract:**
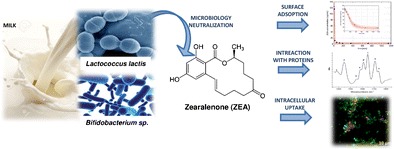
Microbiology neutralization of zearalenone using *Lactococcus lactis* and *Bifidobacterium* sp. was confirmed by identification of binding kinetics and spectroscopic studies such as FT-IR spectroscopy and MALDI-TOF-MS spectrometry. The mechanism of ZEA binding was also investigated.

Microbiology neutralization of zearalenone using *Lactococcus lactis* and *Bifidobacterium* sp. was confirmed by identification of binding kinetics and spectroscopic studies such as FT-IR spectroscopy and MALDI-TOF-MS spectrometry. The mechanism of ZEA binding was also investigated.

## Introduction

**Fig. 1:**
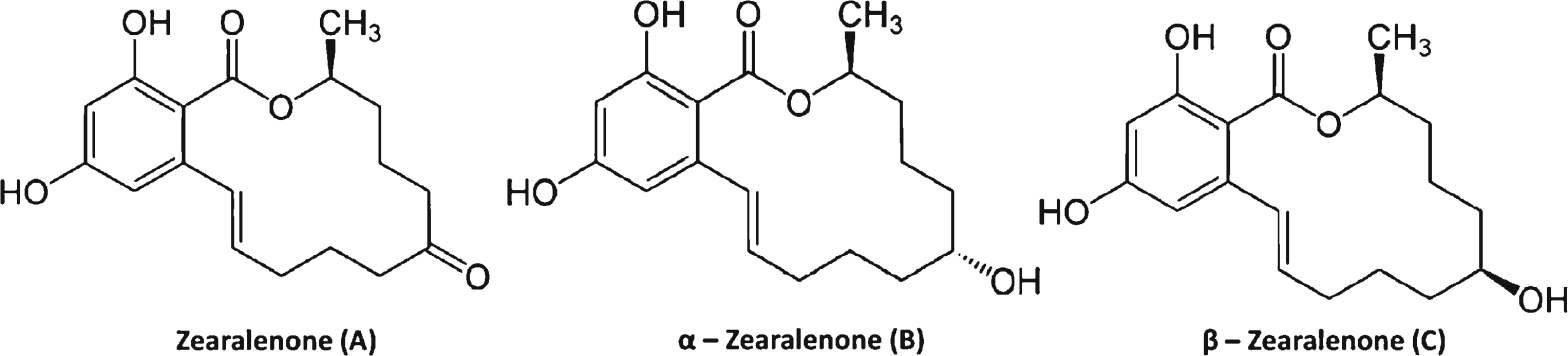
Chemical structures of ZEA and its derivatives: (**A**) zearalenone (ZEA), (**B**) α-zearalenol (α-ZOL), (**C**) β-zearalenol (β-ZOL); based on [6]

The *Fusarium* family is an important cereal pathogen worldwide because of its ability to produce toxic secondary metabolites (mycotoxins) [1]. After infection, mycotoxins can accumulate into cereal plant, resulting in contamination of animal feed and human cereal food in toxicologically relevant concentrations [2]. The main mycotoxins found in agricultural products are aflatoxins (AFs), zearalenone (ZEA), deoxynivalenol, and its derivatives, fumonisins, patulin, and ochratoxin A (OTA) [3]. Zearalenone is produced by *Fusarium* fungi, including *Fusarium graminearum*, *Fusarium culmorum*, *Fusarium cerealis*, *Fusarium equiseti*, and *Fusarium semitectum* [4]. Mostly, this mycotoxin is present in corn, but it can be also found in other important crops such as wheat, barley, sorghum, and rye throughout various countries of the world [5]. Chemically, zearalenone is a resorcyclic acid lactone described as 6-[10-hydroxy-6-oxo-trans-1-undecenyl]-B-resorcyclic acid lactone (Fig. 1a) [6]. ZEA has structural similarity to the natural estrogens, so it can mimic endogenous estrogens, antagonize their activity, change their mechanism of synthesis and metabolism, or interfere with the synthesis of receptor, which contributes to change and neoplastic, i.e., breast cancer or prostate cancer. The biotransformation of ZEA in animals involves the formation of two major metabolites, α-zearalenol and β-zearalenol (α-ZOL and β-ZOL); alpha zearalenol shows higher estrogenicity than ZEA, but β-ZOL is less estrogenic [7, 8]. ZEA derivatives are shown in Fig. 1.

Concerning the importance and diversity of their toxic effects, the occurrence of mycotoxins in foods is potentially dangerous for public health and it is also considered as a major economic problem [9]. Physical and chemical methods have been developed to control the occurrence of these microorganisms and their toxins, but no efficient strategy has yet been proposed to reduce the presence of mycotoxins. There are a few methods which have been developed to control the occurrence of *Fusarium* and their toxins, i.e., physical and chemical approaches [10, 11]. Unfortunately, they are non-efficient and contribute to changes in the value of food products, the organoleptic properties of the purified substance, and the occurrence of toxic substances residues [12]. In the last 10 years, microbiological methods have received much attention [12–21]. They have been found to be safer, more effective, and not inducing harmful side effects that could adversely affect the health and life of humans and animals [22]. One of the most promising organisms able to ZEA neutralization seems to be lactic acid bacteria (LAB). These microorganisms are widely used for the production of fermented foods and are also part of intestinal microflora. Moreover, a lot of reports indicate that LAB have beneficial health effects in humans [22–24]. Recently, researches have been trying to investigate if the lactic acid bacteria, among other unique properties, are able to bind mycotoxins and neutralize them. Some authors reported that specific strains of LABs, such as *Lactobacillus rhamnosus*, *Lactobacillus plantarum*, or *Lactobacillus acidophilus* can reduce concentration of mycotoxins present in food and feed [20, 25, 26]. There are also some reports of neutralization by others microorganism such as *Saccharomyces cerevisiae, Bacillus subtilis, Pseudomonas* sp., or *Rhizopus* strains [14–16, 27]. Therefore, neutralization of zearalenone by lactic acid bacteria could be an interesting alternative to physical and chemical methods because of LAB’s strong antimicrobial properties of produced bacteriocins and bioactive sorption of xenoestrogenes. Fuchs et al. [17] have examined detoxification of patulin (PAT) and ochratoxin A (OTA) by *L. acidophilus* and *Bifidobacterium animalis*. Results of experiment indicated that both strains are highly effective*; L. acidophilus* caused a decrease about 95% of OTA and *Bifidobacterium* reduced the level of PAT by 80%. However, the antifungal activity of lactic strains is still under debates because there is a limited number of reports which have shown results of using LAB in the control of mold growth and neutralization of ZEA [25–27]. Due to this fact, development of new microbiology mycotoxins neutralization method is desirable.

In this paper, a novel approach of zearalenone neutralization by lactic acid bacteria such as *L. lactis* and *Bifidobacterium* sp*.* is investigated. What is more, the physicochemical study of neutralization and mechanism of ZEA binding is described.

## Materials and methods

### Biological material

The two strains of lactic acid bacteria (*L. lactis* and *Bifidobacterium* sp.) were isolated from milk products (Dairy Cooperative in Drzycim, Poland) according to [28]. The LAB strains were cultured in Tryptone Soya Agar (Soybean Casein Digest Medium, Oxoid, Basingstoke, UK) and M9 (Sigma-Aldrich, Warsaw, Poland). Other chemicals, i.e., zearalenone, dimethyl sulfoxide, α-cyano-4-hydroxycinnamic acid, acetonitrile, formic acid, phosphate-buffered saline, ethanol, acridine orange, and ethidium bromide, were obtained from Sigma-Aldrich (Warsaw, Poland).

### Preparation of lactic bacterial cells modified by zearalenone

*L. lactis* and *Bifidobacterium* sp*.* strain were cultured in 150 mL of sterile M9 medium with 6 mL of 10% glucose (final concentration of glucose was 0.4%). The culture of LAB was incubated with shaking at 37 °C for 24 h. Thereafter, 2 ml of each culture at 3.26 McFarland (9.78 × 10^8^ CFU/mL) and 3.67 McFarland (11.01 × 10^8^ CFU/mL) was transferred into new sterile glass tubes. To them, 50 μL of ZEA (in dimethyl sulfoxide) to final concentration 130 μg/mL (C_0_) was added. Probes were incubated with shaking at 37 °C in time intervals 0, 10, 20, 60, 120, 180, 360, 720, 1140, and 1200 min. After mentioned time, the reaction was stopped and optical density (OD) of samples was measured. In order to separate supernatant from bacterial pellet, the samples were centrifuged (4 min, 13,000 rpm). Obtained pellets were used in Fourier transform infrared spectroscopy (FT-IR) and matrix-assisted laser desorption/ionization time-of-flight mass spectrometry (MALDI-TOF-MS) analysis and for the determination of cells viability by fluorescence microscopy. Content of ZEA in supernatants was measured by liquid chromatography electrospray ionization tandem mass spectrometry approach (LC-ESI-MS/MS).

### Fourier transform infrared spectroscopy analysis

FT-IR analysis was performed with a Direct Detect Merck Millipore spectrophotometer (Germany). In total, 2 μL of sample was pressed into a card and dried. All IR spectra were recorded at room temperature in range of 1350–1850 cm^−1^.

### Matrix-assisted laser desorption/ionization with mass spectrometry analysis

Bacterial cells bound by zearalenone were extracted with ethanol and formic acid; bacterial pellet was centrifuged (4 min, 13,000 rpm) and supernatant was decanted. To obtained pellet, 300 μL of water and 900 μL of ethanol were added. All samples were centrifuged (2 min, 13,000 rpm), and the supernatant were separated. Then, 2 μL of 70% formic acid and 2 μL of acetonitrile were added; all samples were centrifuged (2 min, 13,000 rpm) again. In total, 0.75 μL of each sample was spotted on MALDI-TOF-MS MTP AnchorChip 384 in triplicate. For the next spots, 0.75 μL of zearalenone at concentration 5 mg/mL was added (also in triplicate). Next, α-cyano-4-hydroxycinnamic acid (HCCA) matrix at concentration 10 mg/mL was prepared; 1.5 mg of HCCA was dissolved in 150 μL of standard solution (50% ACN, 47.5% H_2_O, and 2.5% TFA). After drying, 0.75 μL of prepared matrix was spotted on each spot and dried again. Samples were analyzed in reflective positive ionization mode in the 400–2800 m/z range. MS spectra were recorded on Ultraflex Extreme II spectrometer with smart beam laser (*λ* = 355 nm, 2 kHz frequency). FlexControl and FlexAnalysis software were used for evaluation of spectrometric data.

### Determination of cells viability after ZEA neutralization

After 60 and 180 min of incubation, acridine orange (*λ*_exc_ = 503 nm, *λ*_em_ = 530/540 nm) and ethidium bromide (*λ*_exc_ = 493 nm, *λ*_em_ = 620 nm) at final concentration of 0.12 and 0.4 μg/mL, respectively, were added to the samples and incubated for 5 min in room temperature. Next, the bacterial suspension was centrifuged (4 min, 4000 rpm) and bacterial pellet were dissolved in phosphate-buffered saline (PBS, 1×). Control (bacterial cells without zearalenone) was also performed. All samples were stored in the dark. Determination of LAB viability after ZEA neutralization was carried out using fluorescence microscope Zeiss Axiocom D1 (Germany); set of filters 43 He and 38 were used. Recorded images were analyzed with Axio Vision 4.8. software.

### High-performance liquid chromatography analysis

The Shimadzu HPLC system (Tokyo, Japan), equipped with a binary solvent delivery system (LC-30AD), SPD-M20A UV diode array detector, autosampler (SIL-30AC), column thermostat (CTO-20AC), and data acquisition station, was used for the chromatographic analyses. For instrument control, data acquisition and processing LabSolution 5.8 software was used.

Zearalenone was separated using ACE C8 column dimensions of 150 × 4.6 mm and 5 μm particle size. The mobile phase consisted of 0.1% formic acid in deionized water and acetonitrile (60:40). The flow rate was 0.6 mL/min, the injection volume was 1 μL, separation temperature was 40 °C, and absorbance was measured at *λ* = 270 nm. The method was validated under optimized conditions. The calibration curve for zearalenone was linear over the concentration range 0.01–1000 μg/mL. The concentration range was obtained with the regression curve (y = ax + b) and correlation coefficient (*R* ^2^) was 0.998. The percentage of ZEA adsorbed by LAB cells was calculated using Eq. (1).

1 $$ E\%=\frac{100^{\ast}\left({C}_0-C\right)}{C_0} $$

where *C*_0_ is an initial concentration of zearalenone and *C* is the concentration of zearalenone at the appointed time. The experimental kinetic data were modeled using the zero-order kinetic model based on the following equation:

2 $$ C={C}_0\hbox{--} {k}^{\ast }t $$

where *C*_0_ is the initial concentration of ZEA expressed in μg/mL, *C* is ZEA concentration (μg/mL) in aqueous phase at time *t*, *t* is the time of zearalenone binding process (min), and *k* (μg/mL min^−1^) is constant rates of the zero-order kinetic model.

## Results

### Kinetic study of ZEA binding to lactic acid bacteria

In order to monitor the kinetic mechanism of ZEA binding to lactic acid bacteria, the concentration of ZEA in the supernatant after biosorption by LAB in the function of time was measured. Results of performed kinetic study are important information for better understanding the factors that influence the rates of the binding process. The obtained experimental kinetic data were tested against the zero-order kinetic models to determine the constant of ZEA uptake rate and to characterize the possible binding mechanism of zearalenone by lactic acid bacteria. To determine the concentration of zearalenone, the high performance liquid chromatography was performed.

**Fig. 2:**
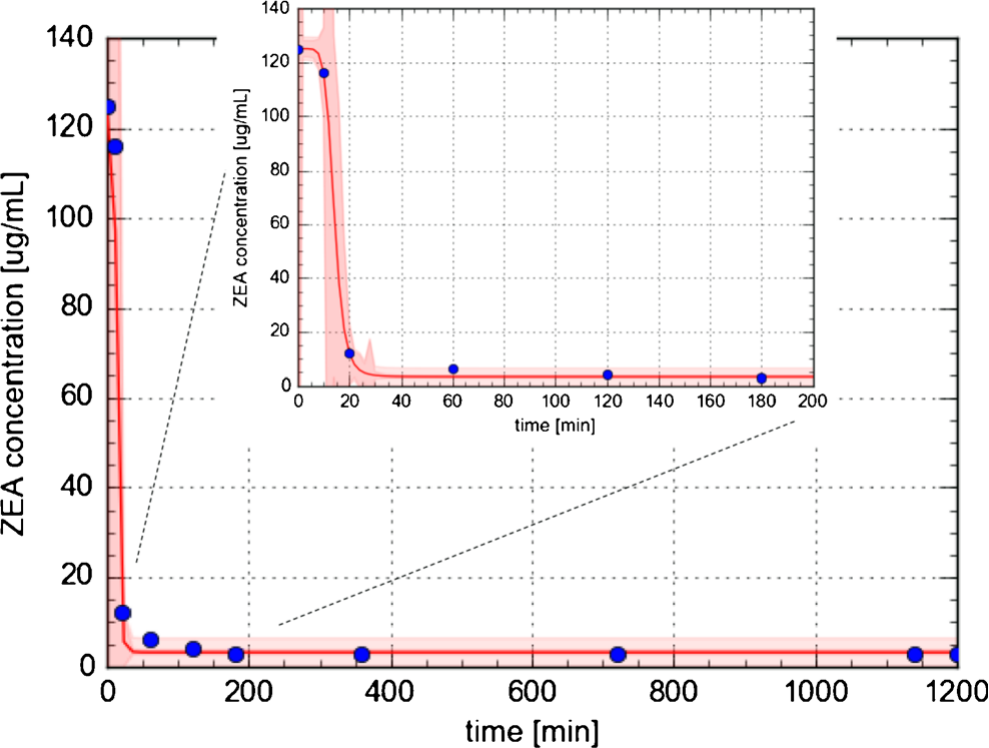
Kinetics of zearalenone binding by *Lactococcus lactis*; standard deviation (SD) is shown as a red shadow

The decrease of ZEA concentration in solution and the efficiency of zearalenone binding to *L. lactis*, as a function of time, have been shown in Fig. 2. The kinetic measurements showed that concentration of zearalenone decreased with increasing incubation time and the kinetic curve was expressed by two different stages: the first one is quite rapid stage of zearalenone biosorption, and the second stage undergo system to equilibrium. Rapid decrease in zearalenone concentration in samples was observed during the first 20 min of the experiment, then the uptake rate of ZEA by *L. lactis* was significantly reduced to finally achieve the equilibrium. About 90% of zearalenone was bound by LAB in the first kinetic stage and additionally about 7% in the second stage. Then the system reached equilibrium. The calculated constant rate of the zero-order kinetic model had the value of 5.49 and 0.15 μg/mL min^−1^ for first and second stage of biosorption process, respectively.

**Fig. 3:**
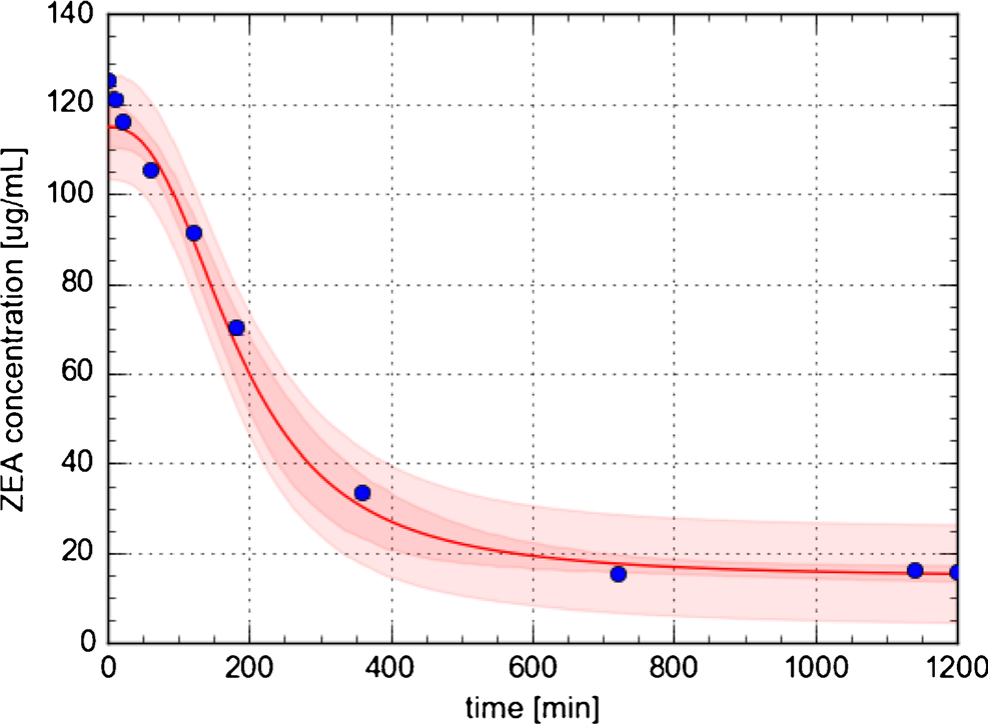
Kinetics of zearalenone binding by *Bifidobacterium* sp*.;* standard deviation (SD) is shown as a red shadow

Neutralization of zearalenone by *Bifidobacterium* sp*.* is also characterized by a significant decrease ZEA concentration in solution in time (Fig. 3), and two stages of biosorption process can be divided. In the first one, lasting first 720 min, effectiveness of biosorption increased rapidly and about 88% of zearalenone was bound to the bacterial cells. After it, the system has reached equilibrium stage. The calculated constant rate of the zero-order kinetic model had the value of 0.75 μg/mL min^−1^ stage of described process. The ZEA biosorption by *Bifidobacterium* sp*.* is homogenous.

### Spectroscopic analysis of lactic acid bacteria after ZEA neutralization in the infrared range

The aim of the FTIR study was to localize active chemical groups of bacterial proteins which contributed to the process of zearalenone uptake by *Lactococcus lactis* and *Bifidobacterium* sp.

**Fig. 4:**
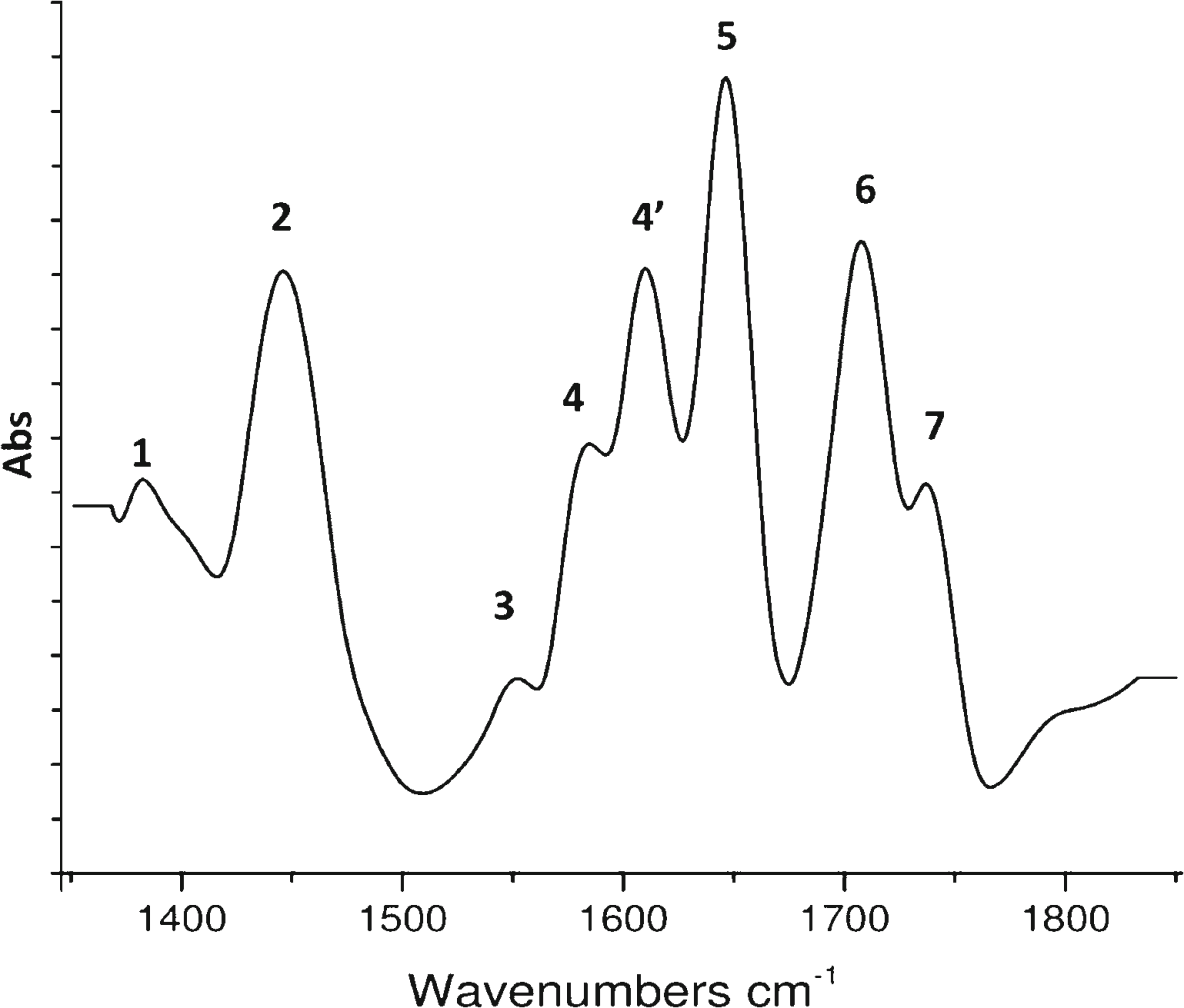
FTIR spectra of zearalenone; *υ* [cm^−**1**^]: *1*: 1375–1385, *2*: 1455–1460, *3*: 1540–1552, *4*: 1580–1590, *4′*: 1600–1615, *5*: 1650–1660, *6*: 1715–1720, *7*: 1735–1745

The obtained FTIR spectra (Fig. 4) of zearalenone showed the presence of spectral bands at *υ* = 1375 cm^−1^ (1) corresponding to the hydroxyl groups. Signal at *υ* = 1455–1460 cm^−1^ (2) derivate from the stretching vibrations of methyl group and the signal at *υ* = 1540–1552 cm^−1^ (3) region from of phenyl ring vibration [29]. There are also peaks at *υ* = 1580–1590 cm^−1^ (4) and 1600–1615 cm^−1^ (4′) corresponding to the ring C–C stretching vibration [30]. Signals at *υ* = 1650–1660 cm^−1^ (5), *υ* =1715 cm^−1^ (6), and *υ* = 1735–1745 cm^−1^ (7) may be attributed to the carbonyl group of ZEA ring. Those results correspond with chemical structure of ZEA; the zearalenone molecule contains two reactive −OH groups on the benzene ring and two less reactive carbonyl groups on the 14-membered macrocyclic lactone ring [6].

**Fig. 5:**
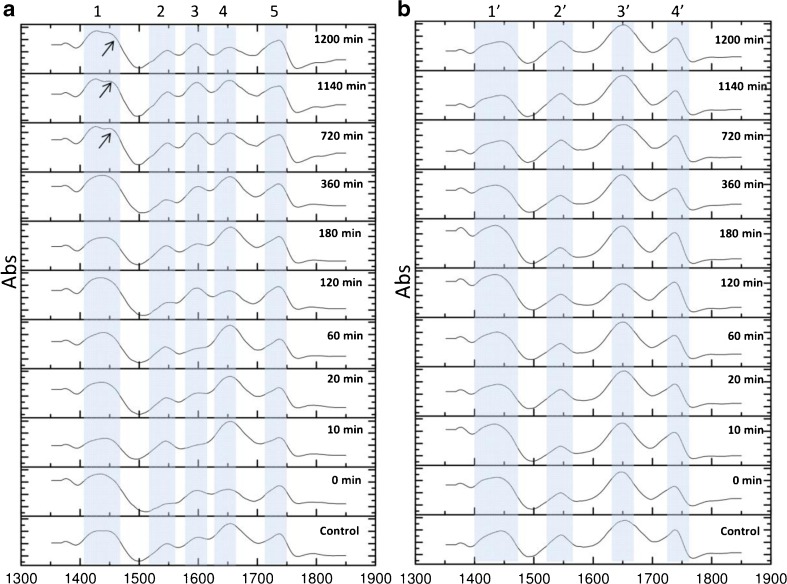
(**A**) FT-IR spectra of *Lactococcus lactis* after zearalenone biosorption; *υ* [cm^−**1**^]: *1*: 1415–1460, *2*: 1520–1560, *3*: 1580–1620, *4*: 1640–1660, *5*: 1715–1750. (**B**) FT-IR spectra of *Bifidobacterium* sp. after zearalenone biosorption; *υ* [cm^−**1**^]: *1′*: 1415–1460, *2′*: 1520–1560, *3′*: 1640–1660, *4′*: 1730–1760

According to the Naumann et al. [20], the analysis of bacteria FTIR spectra was focused on *υ* = 1350–1850 cm^−1^ areas for amides groups. The FT-IR spectra of *L. lactis* after zearalenone neutralization is shown in Fig. 5a. There is a fission of signal at *υ* = 1415–1460 cm^−1^ (1) at incubation time 720, 1140, and 1200 min respectively in comparison with spectral bands at shorter incubation time intervals. What is more, after 720, 1140, and 1200 min of incubation with ZEA, spectral bands at *υ* = 1520–1560 cm^−1^ (2) corresponding to the deprotonated carboxyl groups contribution for amid I on ZEA binding [30]. A signal at *υ* = 1640–1660 cm^−1^ (4) and at *υ* = 1715–1750 cm^−1^ (5) in each sample mean that they derived from stretching vibration of carbonyl groups (C═O) from amid II and III, respectively. Control band at υ(max) = 1600 cm^−1^ is shifted to υ(max) 1590 cm^−1^ (*υ* = 1580–1620; 3) with the increase of ZEA incubation time. It is characterized by signal corresponding to the stretching vibrations of C═C groups in aromatic ring of zearalenone; it indicates the biosorption of ZEA by *L. lactis* and involvement of π–π interactions in uptake process. Furthermore, band at *υ* = 1725 cm^−1^ (5) shows modification of C═O group vibrations caused by influence of zearalenone on bacterial cell.

In comparison with control, the FTIR spectra of *Bifidobacterium* sp*.* after zearalenone binding (Fig. 5b), a signal at *υ* = 1415–1460 cm^−1^ (1′) is changed the band shape. These data correspond to results obtained in *L. lactis* after ZEA neutralization FTIR analysis. There is also spectra band at *υ* = 1520–1560 cm^−1^ (2′); this peak is related with stretching vibrations of C═C group from the aromatic ring of zearalenone and point out occurrence of the biosorption process [30]. Signals at *υ* = 1640–1660 cm^−1^ (3′) and at *υ* = 1730–1760 cm^−1^ (4′) correspond to amides.

### Cells viability after ZEA neutralization

To determine lactic acid bacteria cells after zearalenone biosorption, fluorescence-based cell viability method was chosen. To samples after 60 and 180 min of incubation, acridine orange, which is permeable for live and dead cells, and ethidium bromide (not permeable for live cells) were added. After staining, samples were detected with fluorescence microscopy; live cells exhibit green fluorescence and dead one are visible as a red [31, 32].

**Fig. 6:**
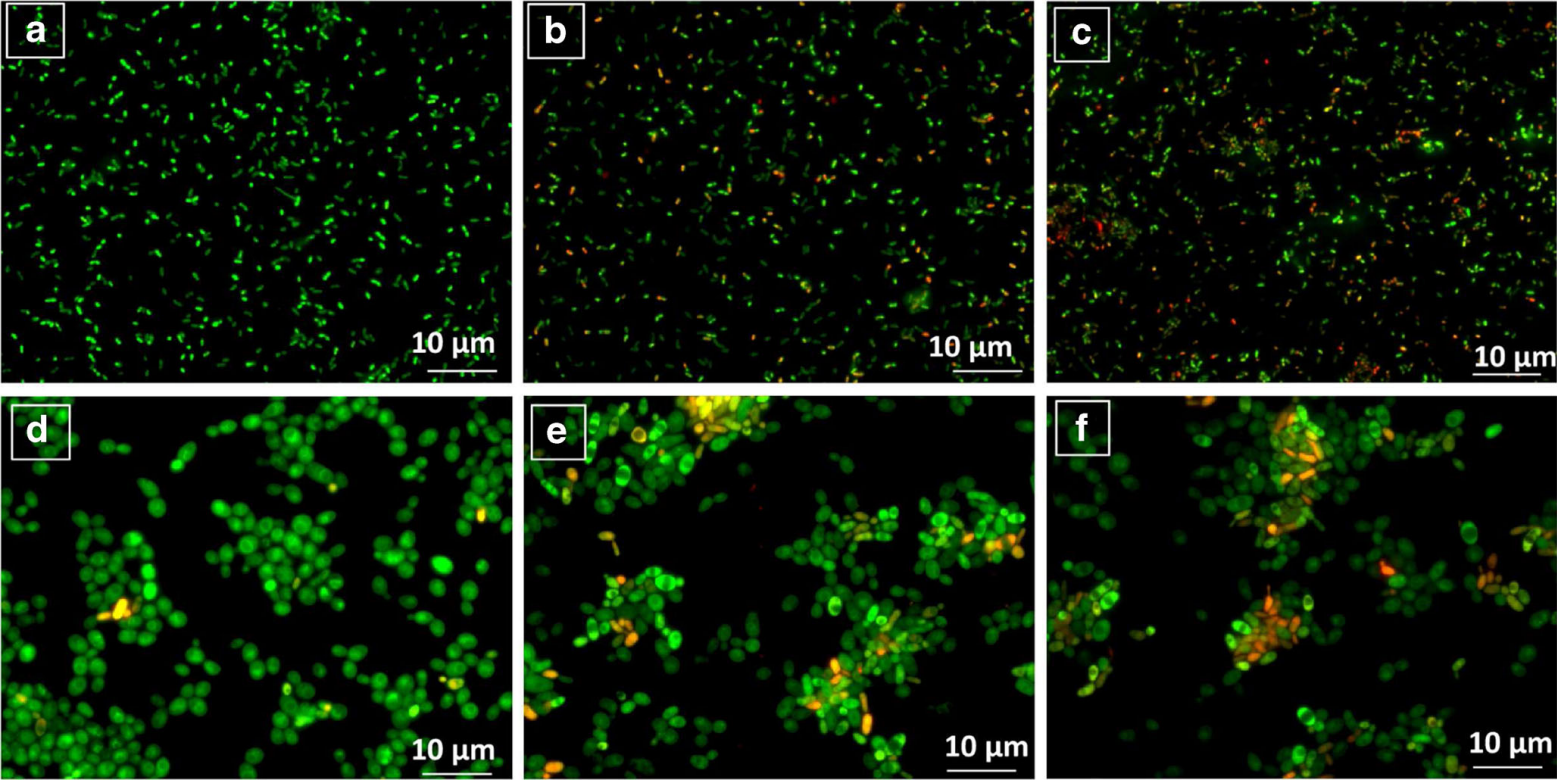
*Lactococcus lactis* cells viability: (**A**) non-incubated with ZEA, (**B**) after 1 h of incubation with ZEA, (**C**) after 3 h of incubation with ZEA and *Bifidobacterium* sp*.* cells viability: (**D**) non-incubated with ZEA, (**E**) after 1 h of incubation with ZEA, (**F**) after 3 h of incubation with ZEA

**Fig. 7:**
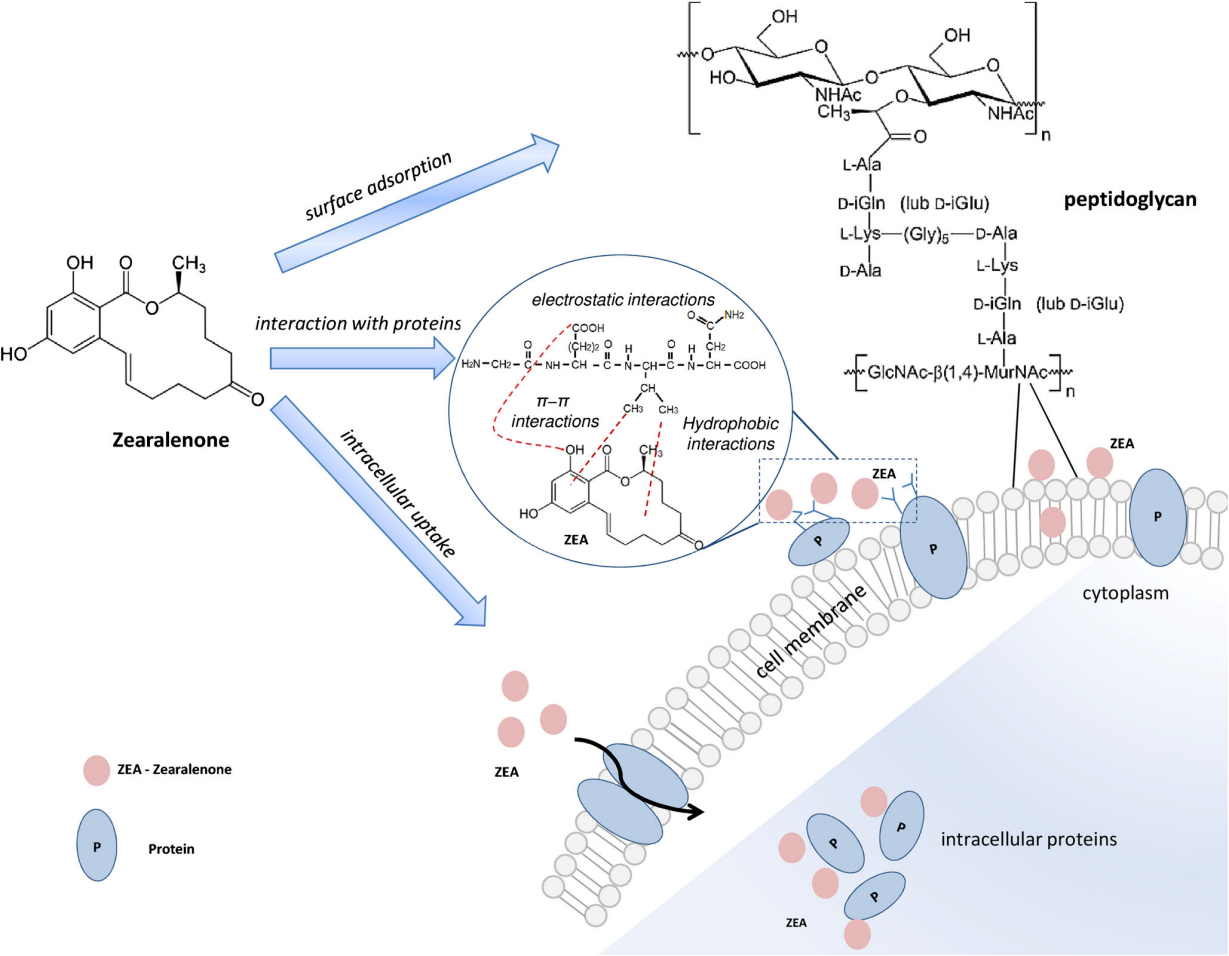
The proposed mechanism of ZEA neutralization by *L. lactis* and *Bifidobacterium* sp*.*; ZEA can be adsorbed at the surface of bacteria and interact with the peptidoglycan or surface proteins or taken into bacterial cell

*L. lactis* cells non-incubated with zearalenone, after 1 and 3 h incubation has been shown in Fig. 6a–c respectively. The fluorescent microscopy analysis indicated that with increasing incubation time, the number of dead bacterial cells is higher. Results of cell viability test for *Bifidobacterium* also showed the same correlation (Fig. 7d–f).

### MALDI-TOF-MS analysis for lactic acid bacteria after ZEA neutralization

The MS spectra were recorded for the HCCA matrix, zearalenone, M9 minimal medium, bacteria not subjected to the process of biosorption (negative control), and bacteria incubated with zearalenone, respectively.

From the table documenting the characteristic m/z peaks of the MALDI-TOF spectrum for *L. lactis* (Table 1), the occurrence of new common signals for different incubation times and characteristic signals only for specific samples can be observed. In sample of bacteria incubated with ZEA for 0, 10, and 20 min, a signal at 854 *m*/*z* is present. Moreover, after 720 min of biosorption, a signal (867 *m*/*z*) not occurring in the others samples appeared. After incubation time extension, peak at 1065 *m*/*z* (for sample incubated for 0, 10, and 20 min) and at 1081 *m*/*z* (10 and 20 min) is disappeared. MALDI results of them have confirmed the presence of zearalenone molecules in the investigated bacteria samples.

**Table 1:**
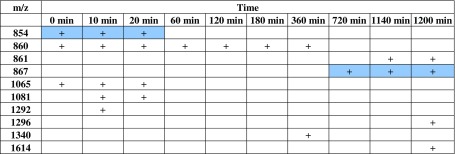
List of characteristic peaks occurring in the MALD-TOF spectrum for *Lactococcus lactis* incubated with zearalenone during the 1200 min; signals mentioned in text are marked with a blue color

According to the results of MALDI-TOF-MS analysis for *Bifidobacterium* sp*.*, an occurrence of new peaks, characteristic only for the specific sample, can be observed (Table 2). Signals 2004, 2290, 2533, and 2788 *m*/*z* are present only in control sample (0 min) without incubation with ZEA. Signal 384 *m*/*z* disappeared after 60 min of incubation. Those results indicate the occurrence of a ZEA neutralization process conducted by lactic acid bacteria.

**Table 2:**
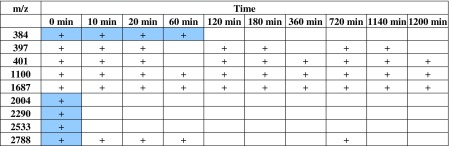
List of characteristic peaks occurring in the MALD-TOF spectrum for *Bifidobacterium sp*. incubated with zearalenone during the 1200 min; signals mentioned in text are marked with a blue color

## Discussion

The occurrence of mycotoxins in foods is dangerous for human health and it is considered as a major economic problem [9], so development of new and effective method of neutralization of zearalenone, an estrogenic compound and inductor of breast and prostate cancer, is required. Results obtained in this study confirmed neutralization of zearalenone by lactic acid bacteria such as *L. lactis* and *Bifidobacterium* sp*.* which is a new and promising approach to the microbiology neutralization of mycotoxins such as ZEA.

Results of kinetic studies indicate the rapid first stage of ZEA binding; in the first 720 min of biosorption by *Bifidobacterium* sp., the bacterial cell wall is saturated by ZEA. During this time, effectiveness of biosorption increased linear and about 88% of zearalenone is bound to the *Bifidobacterium* cells. Another mechanism is characteristic for *L. lactis*, where two different stages of ZEA sorption are observed; the first one is quite rapid (during the first 20 min about 90% of zearalenone were bound by LAB) and the second stage undergo system to equilibrium. Kinetic data are confirmed by constant rate of the zero-order kinetic model and can be related with the literature. In the work of Čvek et al. [25], the capacity of *Lactobacillus rhamnosus and Lactobacillus plantarum* for binding of zearalenone was investigated. The results show that about 98% of initial concentration (20 μg/mL) of ZEA from suspension has been attached to the cells of *L. plantarum* and between about 80% of ZEA has been bounded to the cells of *L. rhamnosus* at the beginning of incubation. After 72 h of incubation, the amount of ZEA in suspension has decreased slightly what can point out release of toxin back to the medium; the binding of zearalenone might be reversible process. Peltonen et al. [18] have tested the efficiency of aflatoxin B1 (AFB1) binding by lactic acid bacteria. Data from quantitation of aflatoxin B1 in supernatant samples by HPLC analysis pointed out that *Lactobacillus*, *Bifidobacterium*, and *Lactococcus* strains are able to binding AFB1 and bound 17.3 to 59.7%, 18.0 to 48.7%, and 5.6 to 41.1% of mentioned mycotoxin, respectively. Moreover, the efficient AFB1 binding by these strains was a rapid process; most of aflatoxin B1 were bound to LAB at first minutes of incubation. Those results correspond to data obtained in our study which also indicate rapid first stage of ZEA biosorption by *Bifidobacterium* sp. (during first 720 min) and *L. lactis* (first 20 min of process). According to Weber-Morris model, the first rapid stage of ZEA sorption (80%) by *L. lactis* strain is limited by surface adsorption, and the second step (7%) is determined by intracellular diffusion of zearalenone [33]. The different sorption mechanism of zearalenone results in different morphology of *Bifidobacterium* sp*.* and *L. lactis*, respectively. Moreover, the *Bifidobacterium* strains have a much more developed surface in comparison with *L. lactis* strains [34, 35]. Basing on the results, it may be concluded that the kinetics of zearalenone binding to lactic acid bacteria consists of distinct stages which correspond to different binding mechanisms. What is more, using a *L. lactis* and *Bifidobacterium* sp*.* strains allowed for the effective adsorption and neutralization of the zearalenone which is the solution to the problem of occurrence of dangerous mycotoxins in the i.e. food.

The proposed mechanism of ZEA neutralization by LAB is shown at the Fig. 7.

LAB are Gram-positive microorganisms with a cell wall which is a complex assemblage of glycopolymers and proteins. LAB cell wall contain about 95% of peptidoglycan—a polymer consisting of sugars and amino acids that forms a mesh-like layer outside the plasma membrane of the bacteria [36]. A major matrix substances in the walls of Gram-positive bacteria are polysaccharides, lipoteichoic (it is bound to the cell membrane by a diacylglycerol), and teichoic acid (bacterial copolymers of glycerol phosphate or ribitol phosphate and carbohydrates) [36–39]. Al components of the Gram-positive cell wall: peptidoglycan, teichoic acids, polysaccharides, and proteins, seem to be crucial in neutralization and binding of zearalenone to the lactic acid bacteria. Peltonen et al. [18] have reported that partial removal of mycotoxins involves physical binding of the toxin probably to the bacterial cell wall or cell wall components. Niderkorn et al. [40] have tested the ability of *Lactobacillus paraplantarum* to bind fumonisins B1 and B2 (FB1, FB2) in fermented foods and feeds; furthermore, they tried to determine the bacterial cell wall component involved in binding mycotoxins. Results of the experiment showed that peptidoglycan and tricarballylic acid (TCA) chains of LAB and FB, respectively, play a significant role in binding interactions; similar components of cell wall were also suggested for the binding of aflatoxin B1 by *L. rhamnosus* [41]. To identify potential functional groups and the possible adsorption sites related to adsorption of zearalenone by *L. lactis* and *Bifidobacterium*, FT-IR analysis was performed. Results of this assay indicate that the main groups involved in this binding process are deprotonated carboxyl groups (spectral bands at *υ* = 1520–1560 cm^−1^) which can derive from both aminoacids (asparagine and glutamine) of bacterial proteins and peptidoglycan of their cell wall [32, 36–39]. Additionally, in biosorption of ZEA, π–π hydrophobic interactions between zearalenone and LAB cells took place [42]. What is more, spectra band at *υ* = 1580–1620 cm^−1^ (Fig. 5a) and *υ* = 1520–1560 cm^−1^ (Fig. 5b) was observed; these peaks are related with stretching vibrations of C═C group from the aromatic ring of zearalenone and point out occurrence of the biosorption process via π–π hydrophobic interaction [6, 43]. To confirm the zearalenone uptake by *L. lactis* and *Bifidobacterium* sp*.*, the MALDI-TOF-MS analysis was conducted. The changes in appearance-disappearance of MS signals in function of time and their characteristic for peptides isotopic pattern suggest the contribution of peptides/proteins in immobilization process of ZEA by LAB. Kinetic data also confirmed the uptake of zearalenone by LABs and may prove the slow penetration of zearalenone into bacterial cells.

The mechanisms by which ZEA damage the cells are not completely understood, but there are some evidences [14, 15, 27] of cytogenic effect and an apoptosis caused by zearalenone. Lioi et al. [44] have examined the induction of chromosome aberrations in in vitro bovine lymphocyte cultures treated with ZEA. It was also recently shown that ZEA increased DNA fragmentation in three cell lines, Vero, Caco-2, and DOK, after 24 h exposure [45, 46]. Despite binding zearalenone to the components of bacterial cell wall, there is possibility of intracellular bioaccumulation and metabolization of ZEA [47–50]. As it is known, the biotransformation of ZEA involves the formation of α-ZOL and β-ZOL, and what is important, α-ZOL shows higher estrogenicity than zearalenone [7, 8]. El-Sharkawy et al. [47] have studied the conversion of zearalenone by various microorganisms and results of their experiment showed that *Streptomyces griseus*, *Streptomyces rutgersensus*, and *Rhizopus arrhizus* were able to metabolize zearalenone to α-zearalenol. In the work of Niderkorn et al. [48], eight *Lactobacilli* and three *Leuconostoc* strains biotransformed ZEA into α-ZOL which point out the capability of lactic acid bacteria to the production of toxic zearalenone derivate. Intracellular uptake of ZEA and then metabolization of it can cause damages of bacterial cells and, in result, leads to death. To determine viability of lactic acid bacteria cells after ZEA neutralization, fluorescent microscopy approach was chosen. Data from this analysis indicated that with increasing incubation time, the number of dead bacterial cells is higher. Red fluorescent of bacterial cells after 1 and 3 h incubation with zearalenone can point out probable loss of cell membrane integrity, DNA damages and even death of *L. lactis* and *Bifidobacterium* cells which can be correlated in some extent with an intracellular accumulation of ZEA. This hypothesis can be confirmed by results from kinetic data where the second stage of ZEA neutralization may prove surface interaction and then the slow penetration of zearalenone into bacterial cells.

## Conclusions

*L. lactis* and *Bifidobacterium* sp*.* strains isolated from milk products have the ability to adsorb and neutralize the zearalenone. Biosorption processes for analyzed bacterial strains are not the same. The sorption process performed by *L. lactis* in comparison with *Bifidobacterium* sp*.* cells is not homogeneous but is expressed with two main stages. The first one is quite rapid and consists of most of zearalenone biosorption (88% for *L. lactis*). The second stage is much slower and corresponds to the diffusion of ZEA into bacterial cells. In case of ZEA uptake process performed by *Bifidobacterium* sp., it is a one-step homogenous process. Results from FTIR analysis indicate that in neutralization of zearalenone by lactic acid bacteria, deprotonated carboxyl group (mainly from asparagine and glutamine) of bacterial proteins and peptidoglycan are mainly involved. According to the shown data, lactic acid bacteria seem to be promising alternatives for the development of new anti-mycotoxin agents.
